# Enhancing Shadow Fading Mitigation with RIS Using Two-Dimensional Orthogonal Pattern Division Multiple Access

**DOI:** 10.3390/s26165225

**Published:** 2026-08-18

**Authors:** Zhiying Yang, Jianguo Yao, Ziwei Liu, Yanling Shi, Xiaodong Bai

**Affiliations:** 1 School of Communications and Information Engineering, Nanjing University of Posts and Telecommunications, Nanjing 210003, China; 1223013836@njupt.edu.cn (Z.Y.); lzw@njupt.edu.cn (Z.L.); ylshi@njupt.edu.cn (Y.S.); 2 School of Computer Science and Technology, Hainan University, Haikou 570228, China; xiaodongbai@hainanu.edu.cn

**Keywords:** orthogonal pattern division multiple access, reconfigurable intelligent surface, multipath effects, Doppler shift, shadow fading

## Abstract

This paper addresses multipath interference introduced by reconfigurable intelligent surface (RIS) in mitigating shadow fading in wireless communication systems. To tackle this, we propose the use of two-dimensional orthogonal pattern division multiple access (OPDMA) with optimal frequency-hopping patterns. OPDMA effectively reduces interference from multipath propagation, Doppler effect, and inter-user interactions, eliminating the need for complex equalization and channel estimation. This simplifies receiver design and enhances system performance. The paper introduces optimal frequency-hopping patterns with ideal autocorrelation and cross-correlation properties, constructed using a two-dimensional cyclic shift based on algebraic Costas arrays. Simulation results demonstrate that the RIS-assisted OPDMA system significantly reduces the bit error rate (BER), improves system throughput, and outperforms existing one-dimensional methods, improving communication quality and system efficiency.

## 1. Introduction

With the rapid evolution of 5G, cellular networks are advancing toward 6G, which must deliver ultra-high data rates, ultra-low latency, and robust performance in complex high-frequency environments. Multipath propagation and Doppler shifts are two major interference sources that challenge reliable communication. Multipath occurs when signals arrive via different paths, causing delays and overlap, while Doppler shifts lead to carrier frequency drift, degrading signal integrity [1]. Developing advanced anti-interference techniques is essential for high-frequency 6G systems.

Orthogonal pattern division multiple access (OPDMA) was introduced in [2] as a multiple access framework that assigns users distinct optimal frequency-hopping patterns over the time–frequency plane. The use of structured time–frequency trajectories distinguishes OPDMA from the resource allocation mechanisms employed by conventional multiple access schemes [3].

Reconfigurable intelligent surface (RIS) technology is widely used to mitigate shadow fading in wireless systems [4]. An RIS is a passive device that optimizes signal propagation by adjusting the phase and amplitude of reflected signals. Rather than actively transmitting, it reflects signals to enhance coverage and mitigate shadow fading while maintaining low power consumption and cost [5].

However, RIS integration introduces additional challenges. The increased number of signal paths leads to complex multipath interference [6,7], requiring precise channel estimation and reflection control. Existing studies have extensively explored RIS-assisted multiple access schemes, which can be classified by their resource allocation dimensionality: as a one-dimensional multiple access technology that allocates resources only in a single dimension (frequency-, time-, or code-domain), traditional orthogonal multiple access (OMA) and its RIS-assisted schemes (RIS-FDMA, RIS-TDMA, RIS-CDMA) can enhance single-user gain via beamforming, but their spectral efficiency is limited by orthogonal resource allocation, with severe beam leakage interference in multi-user scenarios [8,9,10]; as another one-dimensional multiple access technology that distinguishes users only in the power domain, RIS-assisted non-orthogonal multiple access (NOMA) can optimize power multiplexing to improve capacity, but suffers from high successive interference cancellation (SIC) complexity, error propagation, and user fairness imbalance [11]. This paper proposes leveraging OPDMA in RIS-assisted wireless systems, utilizing the ideal autocorrelation properties of optimal frequency-hopping patterns that span the time and frequency dimensions with ideal autocorrelation properties to automatically mitigate multipath interference. Compared to traditional access schemes, RIS-assisted OPDMA improves robustness against multipath and the Doppler effect while reducing implementation complexity. By eliminating the need for complex equalization and simplifying channel estimation, this approach enhances efficiency, making it a promising solution for future 6G deployments.

Current RIS-aided communication studies mainly focus on mitigating natural channel fading and inherent multipath interference. However, RIS deployment produces additional reflective multipaths distinct from traditional channel multipaths, leading to signal distortion and complicated channel estimation. To tackle this problem, this paper adopts a two-dimensional OPDMA scheme with optimized Costas array frequency-hopping patterns. Leveraging the inherent multipath immunity of OPDMA, the proposed scheme effectively suppresses RIS-induced extra multipath interference, and ensures reliable transmission in dynamic scenarios. (i) A two-dimensional OPDMA framework is integrated into an RIS-assisted communication system, in which the RIS enhances shadowed links while OPDMA addresses the additional propagation paths introduced by RIS reflection. (ii) Welch–Costas frequency-hopping patterns generated through two-dimensional cyclic shifts are adapted to the RIS-assisted system, and the delay and Doppler conditions required to preserve pattern orthogonality are specified. (iii) The BER and throughput of the proposed scheme are evaluated against those of RIS-FDMA, RIS-TDMA, RIS-CDMA, and RIS-NOMA under a common simulation setting.

The paper is structured as follows: Section 2 introduces RIS principles, focusing on signal optimization through reflection. Section 3 discusses constructing Welch–Costas arrays using finite field theory, analyzing their autocorrelation properties, and generating frequency-hopping patterns. Section 4 examines the design and analysis of optimal frequency-hopping patterns based on Welch–Costas arrays. Section 5 evaluates the BER and throughput performance of the proposed RIS-assisted two-dimensional OPDMA scheme via extensive simulations, comparing it against conventional one-dimensional multi-access techniques including FDMA, TDMA, CDMA and NOMA. Finally, Section 6 summarizes the contributions and findings.

## 2. Overview of Reconfigurable Intelligent Surface Technology and Applications

The RIS mitigates shadow fading, thereby reducing reliance on costly infrastructure expansion. By dynamically adjusting the signal path via its reflecting elements, the RIS redirects obstructed signals to the receiver, optimizing transmission efficiency [12,13].

To realize the low-cost and large-scale deployment of wireless communication systems, a 2-bit phase quantization scheme is adopted for the RIS in this paper. Based on the 2-bit quantization characteristics, each RIS meta-atom produces $2^{2}=4$ discrete phase states uniformly distributed within the [0,2π) phase range, which fully covers the phase modulation range of electromagnetic waves. The quantized phase set is defined as $\theta_{i}\in \left\{0,\frac{\pi }{2},\pi ,\frac{3\pi }{2}\right\}$.

In an RIS-assisted OPDMA system, dynamic reflection significantly enhances interference resistance [14,15]. The total channel gain is expressed as follows:

(1) $$h_{\mathrm{eff}}=\sum_{i=1}^{N_{\mathrm{RIS}}}\beta_{i}g_{i}h_{\mathrm{RU},i}e^{j\theta_{i}}+h_{\mathrm{LOS}}+h_{\mathrm{NLOS}}$$

where $h_{\mathrm{eff}}$ is the equivalent channel gain; $h_{\mathrm{LOS}}$ and $h_{\mathrm{NLOS}}$ denote the LOS and NLOS channel components, respectively; $\beta_{i}$ and $\theta_{i}$ are the reflection amplitude coefficient and phase shift of the *i*-th RIS element, with $0\leq \beta_{i}\leq 1$; $g_{i}$ and $h_{\mathrm{RU},i}$ denote the channel gains from the transmitter to the *i*-th RIS element and from that element to the receiver, respectively, both accounting for distance-dependent path loss and fading; and j is the imaginary unit.

To refine the system framework and clarify the complete transmission mechanism, this paper supplements a rigorous end-to-end signal model. The base station transmits OPDMA symbols modulated by optimized two-dimensional Costas frequency-hopping patterns. After propagating through the above equivalent fading channel and incorporating additive noise, the received signal at the user side can be expressed as follows:

(2) $$r(t)=h_{\mathrm{eff}}\cdot x\left(t\right)+w(t)$$

where r(t) is the final received signal, x(t) represents the transmitted two-dimensional OPDMA frequency-hopping signal, and w(t) denotes zero-mean additive white Gaussian noise (AWGN).

When the RIS is employed to enhance the received signal and mitigate shadow fading, let there be $N_{\mathrm{RIS}}$ controllable reflecting elements. For the *i*-th ($i=1,2,\ldots ,N_{\mathrm{RIS}}$) element, denote the delay weighting at time slot k=1 (corresponding to frequency $f_{0}+f\left(1\right)F_{b}$) as $\tau_{i,1}^{\mathrm{RIS}}$, and the delay weighting at slot *k* as $\tau_{i,k}^{\mathrm{RIS}}$. Here, $\tau_{i,k}^{\mathrm{RIS}}$ denotes the propagation delay introduced by the *i*-th RIS element at the *k*-th hopping frequency. To maintain the same phase compensation for different hopping frequencies, we have $\left(f_{0}+f\left(1\right)F_{b}\right)\cdot \tau_{i,1}^{\mathrm{RIS}}=\left(f_{0}+f\left(k\right)F_{b}\right)\cdot \tau_{i,k}^{\mathrm{RIS}}$. Rearranging yields $\tau_{i,k}^{\mathrm{RIS}}=\frac{f_{0}+f\left(1\right)F_{b}}{f_{0}+f\left(k\right)F_{b}}\;\tau_{i,1}^{\mathrm{RIS}}$. The corresponding RIS phase weighting can then be determined from the obtained propagation delay. Furthermore, the relationship of phase weighting with frequency-hopping pattern is given by

(3) $$\theta_{i,k}=\frac{f_{0}+f\left(1\right)F_{b}}{f_{0}+f\left(k\right)F_{b}}\cdot \theta_{i,1}$$

where $f_{0}$ denotes the initial carrier frequency before frequency-hopping is applied. The term $f_{0}+f\left(k\right)F_{b}$ represents the center frequency of the transmitted signal at the *k*-th hopping slot, where k=1,2,…,n; f(k) is the position function of the *n*-th-order Costas sequence, *n* is the order of the corresponding Welch–Costas sequence, and $F_{b}$ denotes the frequency-hop spacing. Therefore, the RIS phase weighting is adjusted according to the hopping frequency at each transmission slot.

In modern communication systems, base stations, relays, and reconfigurable intelligent surface each possess distinct characteristics.

Table 1 presents a comparative analysis of these three technologies, emphasizing the advantages of RIS. Compared to base stations and relays, RIS provides substantial benefits in terms of low power consumption, cost-effectiveness, and high flexibility, particularly in extending network coverage and minimizing infrastructure costs.

## 3. Construction of Welch–Costas Arrays

Costas arrays were introduced for frequency-hopping waveform design [16,17,18]. Finite field theory subsequently enabled algebraic constructions, including Welch– and Golomb–Costas arrays [19,20]. This section presents the Welch construction used to generate the frequency-hopping patterns in Section 4.

Let $F_{p}$ denote the finite field of prime order *p*. Its multiplicative group contains φ(p−1) primitive elements, where φ(·) is Euler’s totient function. Let α be a primitive element of $F_{p}$, define n=p−1, and let $P_{n}$ be a permutation matrix of order *n*.

According to [21], $P_{n}$ forms a Welch–Costas array if and only if its associated placement function satisfies

(4) $$y\left(k\right)\equiv \eta \cdot \alpha^{k}\;\left(\mathrm{mod}\;p\right),\;1\leq k\leq p-1$$

where *k* represents the horizontal coordinate and y(k) represents the corresponding vertical coordinate. By traversing all possible values of *k*, the corresponding coordinate pairs can be obtained, from which a permutation matrix of order *n* is constructed.

Euler’s totient function, φ(n), is calculated using the following formula:

(5) $$\varphi \left(n\right)=n\left(1-\frac{1}{p_{1}}\right)\left(1-\frac{1}{p_{2}}\right)\cdots \left(1-\frac{1}{p_{s}}\right)$$

Here, the positive integer *n* is multiplied by each factor $\left(1-\frac{1}{p_{i}}\right)$, where $p_{1},p_{2},\ldots ,p_{s}$ are the distinct prime factors of *n*.

The prime factorization of *n* is given by the following:

(6) $$n=p_{1}^{e_{1}}p_{2}^{e_{2}}\cdots p_{s}^{e_{s}}$$

This represents *n* as a product of prime factors, where $p_{1},p_{2},\ldots ,p_{s}$ are the primes, and $e_{1},e_{2},\ldots ,e_{s}$ are their corresponding positive integer exponents. This factorization is required to compute φ(n).

From Equations (5) and (6), it follows that the finite field $F_{7}$ has two primitive elements: $\alpha_{1}=3$ and $\alpha_{2}=5$. According Equation (4), the following Welch–Costas arrays can be obtained:

(7) $$y_{1}=\left\{2,3,1,5,4,6\right\}$$

(8) $$y_{2}=\left\{5,4,6,2,3,1\right\}$$

**Figure 1:**
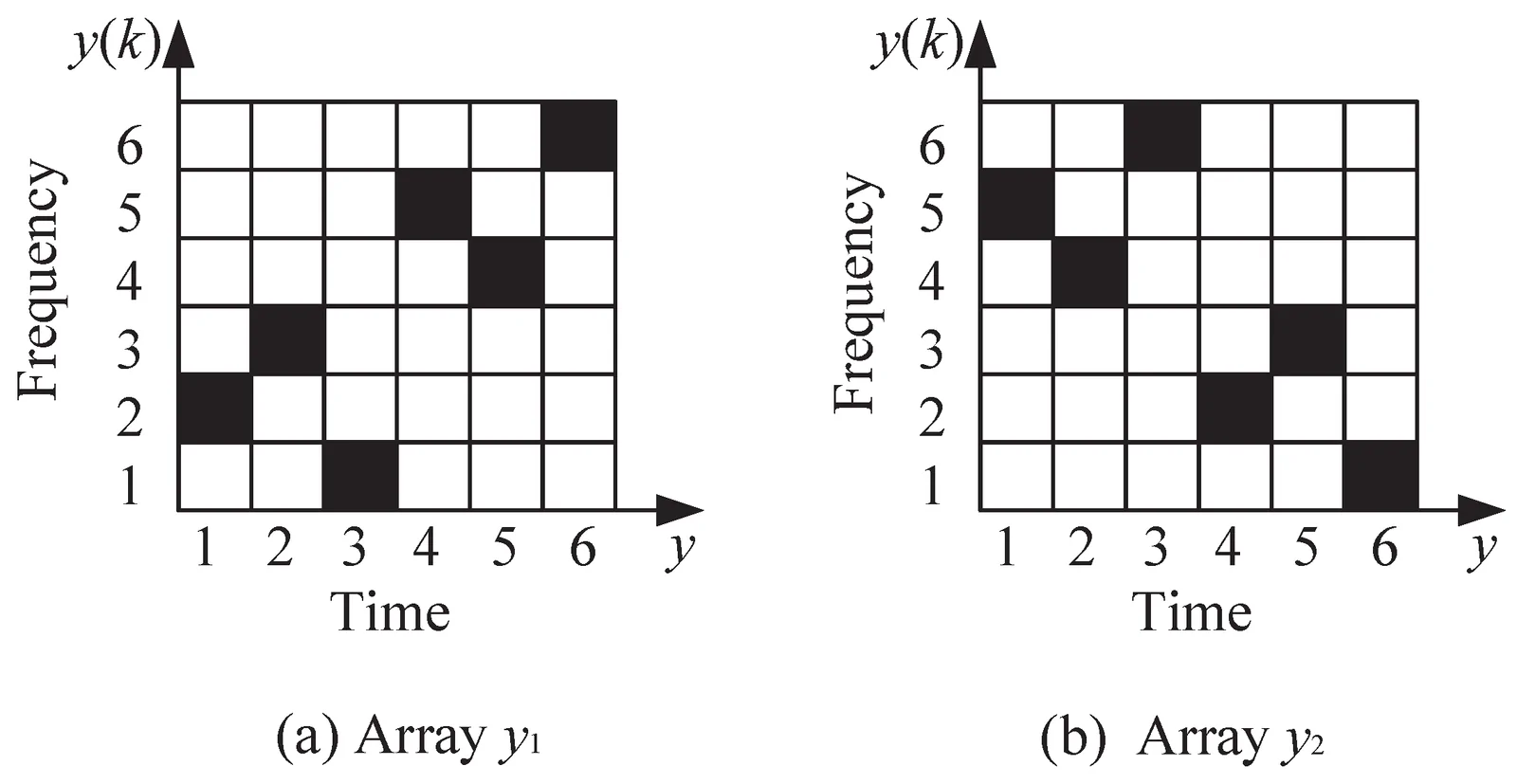
Welch–Costas arrays.

In the Cartesian coordinate system, the horizontal axis corresponds to time, while the vertical axis corresponds to frequency. In the *i*-th column, black squares represent “1” in row y(k), while white squares represent “0”. Figure 1 illustrates the arrays $y_{1}$ and $y_{2}$.

**Figure 2:**
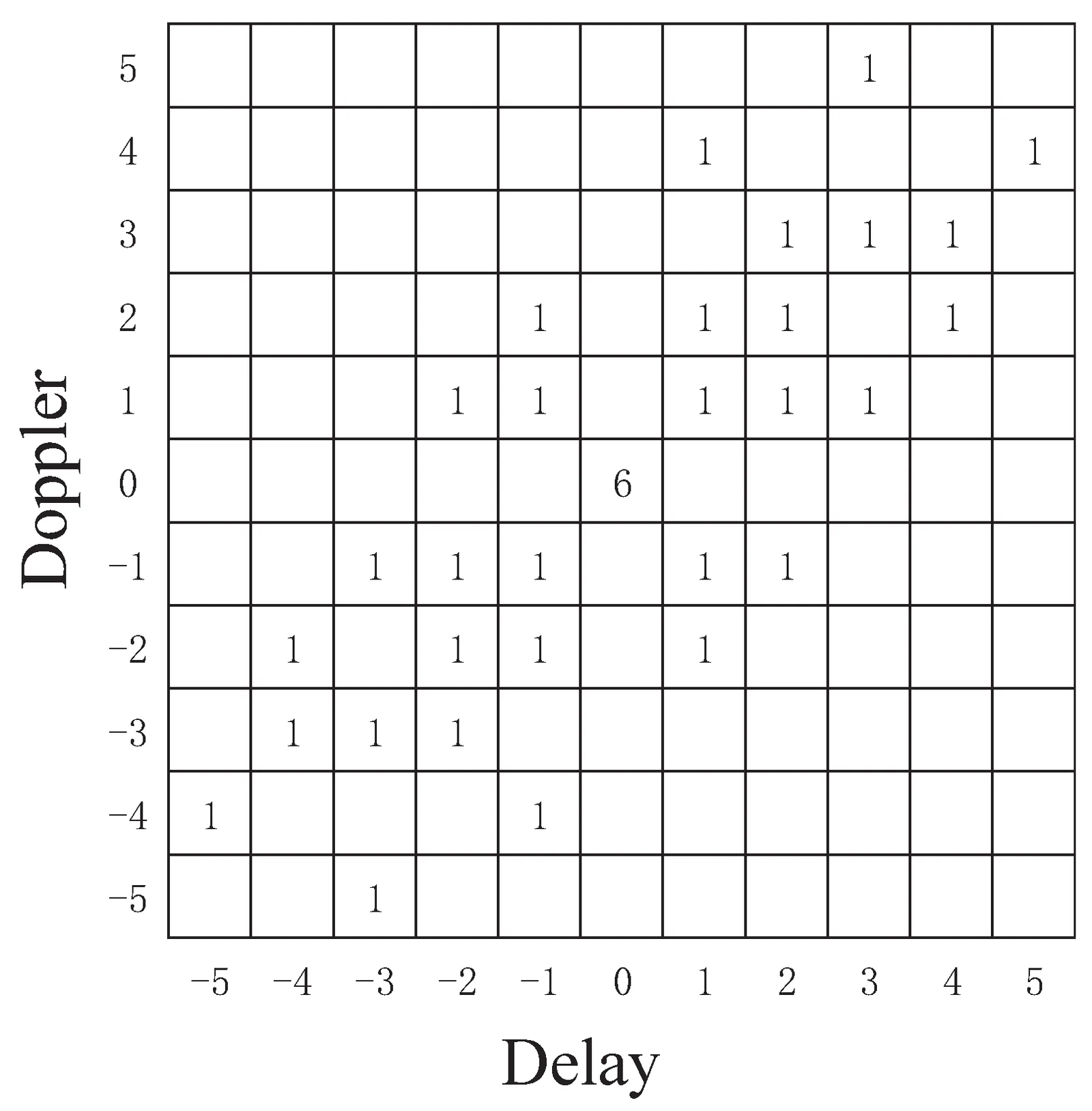
Autocorrelation function of the constructed Welch–Costas array.

The autocorrelation function of the Welch–Costas array $y_{1}$ is shown in Figure 2. Except for the center peak of 6, which corresponds to the array length, all autocorrelation sidelobes have values of either 0 or 1. Therefore, the maximum autocorrelation sidelobe value is 1, indicating that the Welch–Costas array possesses ideal autocorrelation properties.

## 4. Construction of Welch–Costas Array-Based Optimal Frequency-Hopping Patterns

**Figure 3:**
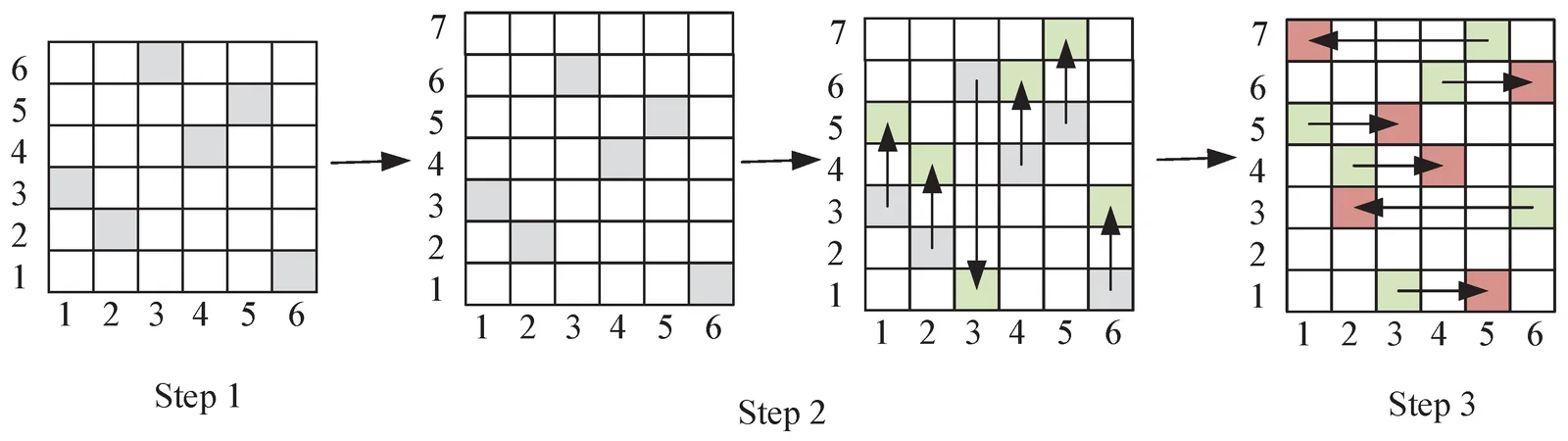
Construction of the optimal frequency-hopping patterns.

The optimal frequency-hopping patterns constructed from carefully designed arrays provide two-dimensional orthogonality in the time and frequency domains. This unique property enables reliable signal transmission over complex wireless channels and effectively mitigates interference among multiple users. The overall procedure for constructing the Welch–Costas array-based optimal frequency-hopping patterns is illustrated in Figure 3.

### 4.1. Step 1: Determine the Order of the Welch–Costas Array and Generate the Initial Array

Consider a cellular cluster consisting of *M* cells. The number of optimal frequency-hopping pattern families, denoted by *N*, should be selected such that N≥M. The maximum Doppler shift $D_{\max}$ is normalized with respect to the frequency-hopping interval, and Δd denotes the minimum Doppler distance between any two optimal frequency-hopping pattern families. To preserve orthogonality in the presence of Doppler effects, the following constraint must be satisfied:

(9) $$0\leq D_{\max}<\Delta d.$$

Assume that each cell supports up to *l* users. The number of frequency-hopping patterns contained in each family, denoted by $N_{1}$, must satisfy $N_{1}\geq l$. Similarly, the maximum multipath delay $\tau_{\max}$ is normalized with respect to the frequency-hopping time slot, and Δτ denotes the minimum delay distance between optimal frequency-hopping patterns within the same family. Therefore, the delay constraint is given by the following:

(10) $$0\leq \tau_{\max}<\Delta \tau .$$

The prime number *p* is then selected according to

(11) $$p\geq \max(N\cdot \Delta d,N_{1}\cdot \Delta \tau +1).$$

Let α be a primitive element of $F_{p}$. A Welch–Costas array can be represented by a permutation matrix P of order n=p−1, whose associated placement function is defined as

(12) $$y\left(k\right)=\alpha^{k},\;k=1,2,\ldots ,n.$$

### 4.2. Step 2: Generate the First Optimal Frequency-Hopping Pattern in Each Family

An empty row is inserted above the *n*-th row of the generated Welch–Costas array P, extending the original array to an (n+1)×n matrix. The resulting matrix is then vertically cyclically shifted by 0,Δd,2Δd,…,(N−1)Δd rows. Consequently, *N* arrays, denoted by $P_{1},P_{2},\ldots ,P_{N}$, are obtained, each containing one empty row. The corresponding placement function of the *i*-th array is given by

(13) $$y_{i}\left(k\right)=\alpha^{k}+\left(i-1\right)\Delta d,\;i=1,2,\ldots ,N.$$

### 4.3. Step 3: Generate the Remaining Optimal Frequency-Hopping Patterns in Each Family

The remaining frequency-hopping patterns within each optimal family are obtained by applying horizontal cyclic shifts to the first pattern generated in Step 2. Specifically, the first pattern of each family is horizontally shifted by $\Delta \tau ,2\Delta \tau ,\ldots ,(N_{1}-1)\Delta \tau$ columns, resulting in $N_{1}-1$ additional patterns.

The placement function of the $n_{j}$-th optimal frequency-hopping pattern in the *j*-th family is given by

(14) $$y_{j,n_{j}}\left(k\right)=\alpha^{\left(n_{j}-1\right)}\cdot \alpha^{k}+\left(j-1\right)\Delta d,\;1\leq k\leq p-1.$$

where j=1,2,…,N and $n_{j}=1,2,\ldots ,N_{1}$. The first pattern in each family corresponds to $n_{j}=1$.

The constructed optimal frequency-hopping patterns exhibit ideal cross-correlation properties provided that the selected Doppler distance exceeds the maximum Doppler shift in the system or the selected delay distance exceeds the maximum multipath delay in the considered cell.

## 5. Simulation Results and Analysis of a RIS-Assisted Two-Dimensional OPDMA System

This section evaluates the BER and effective throughput of the proposed RIS-assisted OPDMA system under the channel and simulation settings described below.

The received signal of user *u* at time instant *t* is expressed as

(15) $$\begin{array}{cc} r_{u}\left(t\right)= & \sqrt{P_{t}}\;h_{\mathrm{eff},u}\left(t,\Theta \right)x_{u}\left(t,f_{u}\left(t\right)\right)\\ \; & +\sum_{i\in I_{t}}\rho_{i}h_{\mathrm{eff},i}\left(t,\Theta \right)x_{i}\left(t-\tau_{i},f_{i}\left(t-\tau_{i}\right)\right)\\ \; & \;\cdot \;\delta \left(f_{i}\left(t-\tau_{i}\right)+d_{i}-f_{u}\left(t\right)\right)+w_{u}\left(t\right). \end{array}$$

In Equation (15), $P_{t}$ denotes the transmit power, and $x_{u}\left(t,f_{u}\left(t\right)\right)$ represents the quadrature phase shift keying(QPSK) signal transmitted by user *u* over the time-varying hopping frequency $f_{u}\left(t\right)$. The term $h_{\mathrm{eff},u}\left(t,\Theta \right)$ denotes the RIS-assisted equivalent channel coefficient of user *u*, which depends on the RIS phase-control matrix $\Theta =\mathrm{diag}\left(e^{j\theta_{1}},e^{j\theta_{2}},\ldots ,e^{j\theta_{N_{\mathrm{RIS}}}}\right)$. Each RIS element adopts a unit-amplitude reflection coefficient, and its phase shift satisfies the 2-bit discrete phase constraint $\theta_{m}\in \left\{0,\frac{\pi }{2},\pi ,\frac{3\pi }{2}\right\}$, where $m=1,2,\ldots ,N_{\mathrm{RIS}}$. By optimizing the discrete phase shifts in Θ, the reflected signal components are combined as coherently as possible to enhance the desired signal.

The second term represents the frequency-collision interference caused by interfering users or multipath components. Here, $I_{t}$ denotes the set of potential interference components at time *t*, while $\rho_{i}$, $\tau_{i}$, and $d_{i}$ denote the amplitude gain, time delay, and Doppler shift of the *i*-th interference component, respectively. The term $h_{\mathrm{eff},i}\left(t,\Theta \right)$ represents the corresponding RIS-assisted equivalent channel coefficient. The indicator function δ(·) equals 1 only when the delayed and Doppler-shifted interference signal occupies the same hopping frequency as user *u*; otherwise, it equals 0. Finally, $w_{u}\left(t\right)$ denotes the complex additive white Gaussian noise (AWGN) at the receiver of user *u*.

Owing to the ideal autocorrelation and cross-correlation properties of the proposed OPDMA hopping patterns, RIS-induced delayed or Doppler-shifted interference components are less likely to occupy the same hopping frequency as the desired signal. Consequently, the interference term is effectively suppressed, resulting in a lower BER.

The average BER of the system is defined as shown in Equation (16).

(16) $${\mathrm{BER}}_{\mathrm{sys}}=\frac{\sum_{u=1}^{U}\sum_{t\in T_{u}}I\left[{\hat{b}}_{u}\left(t\right)\neq b_{u}\left(t\right)\right]}{\sum_{u=1}^{U}N_{u}}$$

where *U* denotes the total number of users in the system, $T_{u}$ represents the set of effective transmission time slots of user *u*, and $N_{u}$ is the total number of effective transmitted bits. $b_{u}\left(t\right)$ and ${\hat{b}}_{u}\left(t\right)$ are the transmitted bit and detected bit of user *u*, respectively, and I[·] is the indicator function.

The total effective throughput of the system is given by Equation (17).

(17) $$\eta_{\mathrm{sys}}=\sum_{u=1}^{U}R_{u}\left(1-{\mathrm{BER}}_{u}\right)$$

where $R_{u}$ is the original transmission rate of user *u*, and ${\mathrm{BER}}_{u}$ stands for the bit error rate of a single user. The BER reduction achieved by suppressing RIS-induced multipath interference consequently increases the effective system throughput according to (17).

### 5.1. Simulation Parameters

The RIS is deployed between the base station and users. All benchmark schemes share the same deployment geometry and user locations to ensure a fair comparison.

The effectiveness of the optimal frequency-hopping pattern design in multi-user communication systems, particularly in complex channel environments, is further evaluated through simulation. All links follow the same distance-dependent path loss model. For fair comparison, the corresponding path loss coefficients are normalized with respect to the reference transmission distance. Small-scale fading follows the Rayleigh distribution, while large-scale fading is modeled by log-normal shadowing. For fair comparison, all users are assumed to have the same RIS–user distance (50 m), resulting in identical RIS gain across all schemes. In practice, RIS hardware impairments, such as mutual coupling and phase quantization errors, may reduce the achievable beamforming gain and degrade the BER and throughput performance. However, they do not affect the orthogonality of the proposed two-dimensional OPDMA hopping patterns, which are determined by the hopping pattern design. The investigation of practical RIS hardware impairments is left for future work. For RIS-NOMA, users are ordered according to their effective channel gains, and users with weaker channel gains are assigned larger power-allocation coefficients. The power-allocation coefficients are determined by solving $\max_{a_{u}}\sum_{u=1}^{U}R_{u}$, subject to the total power and non-negativity constraints, i.e., $\sum_{u=1}^{U}a_{u}=1$ and $a_{u}\geq 0$, where $a_{u}$ and $R_{u}$ denote the power-allocation coefficient and achievable rate of user *u*, respectively. Ideal successive interference cancellation is assumed, meaning that previously decoded user signals are perfectly removed without residual interference or error propagation. Table 2 presents the parameters used to analyze and demonstrate the performance of the OPDMA system in such environments [22].

### 5.2. BER Performance Evaluation

**Figure 4:**
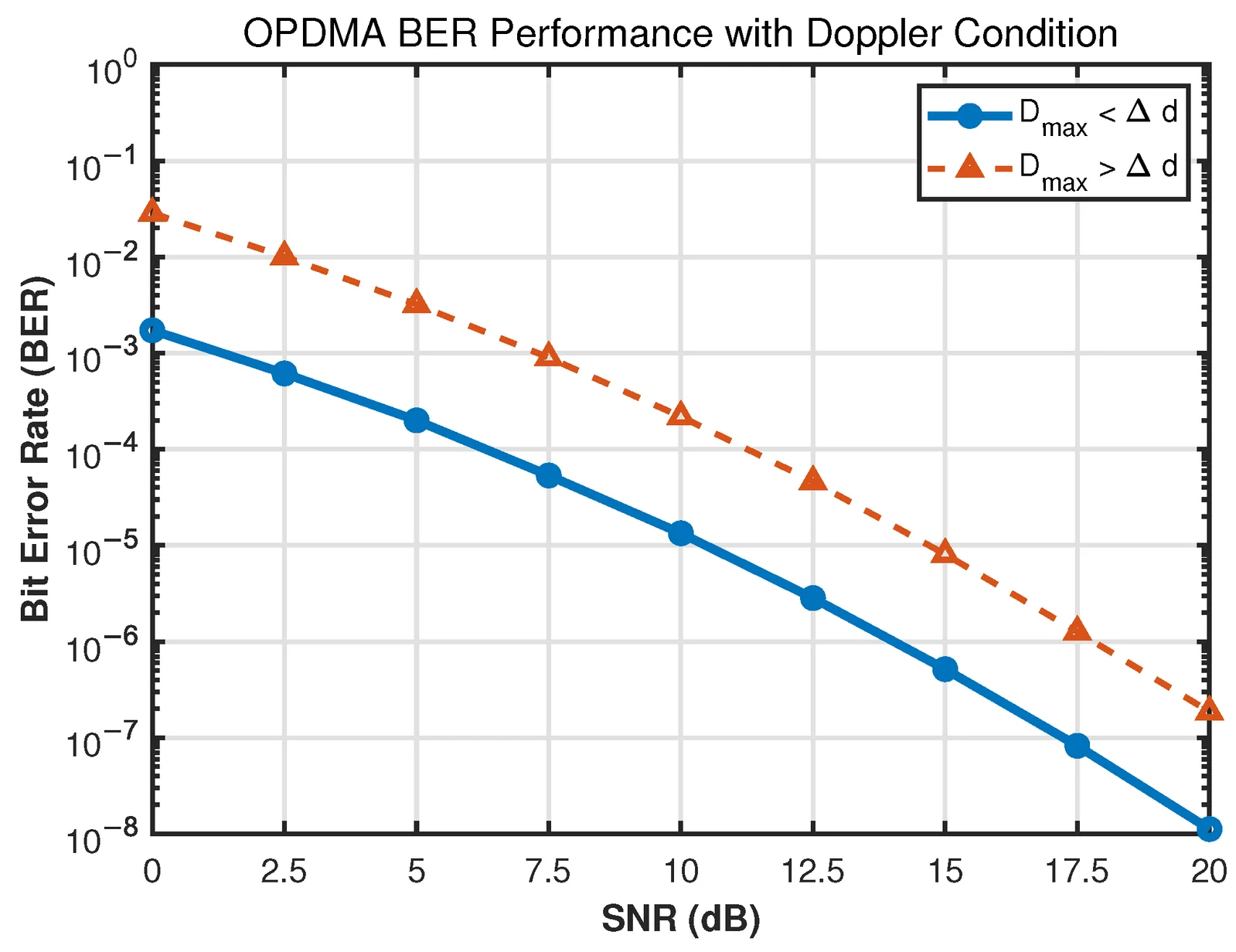
BER of the OPDMA system.

Figure 4 shows the BER simulation results, where a higher SNR reduces the BER, improving system performance. When the maximum Doppler shift $D_{\max}$ is smaller than the minimum shift Δd between frequency-hopping patterns, ideal cross-correlation is maintained, leading to lower BER. However, when $D_{\max}$ exceeds Δd, the cross-correlation deteriorates, resulting in a higher BER.

Under the same conditions as those presented in Table 2, shadow fading, which follows a log-normal distribution with a mean of 0 and a standard deviation of 8 dB, is introduced into the channel. The BER performance of the OPDMA scheme is simulated and compared with those of FDMA, TDMA, CDMA and NOMA under varying SNR levels.

**Figure 5:**
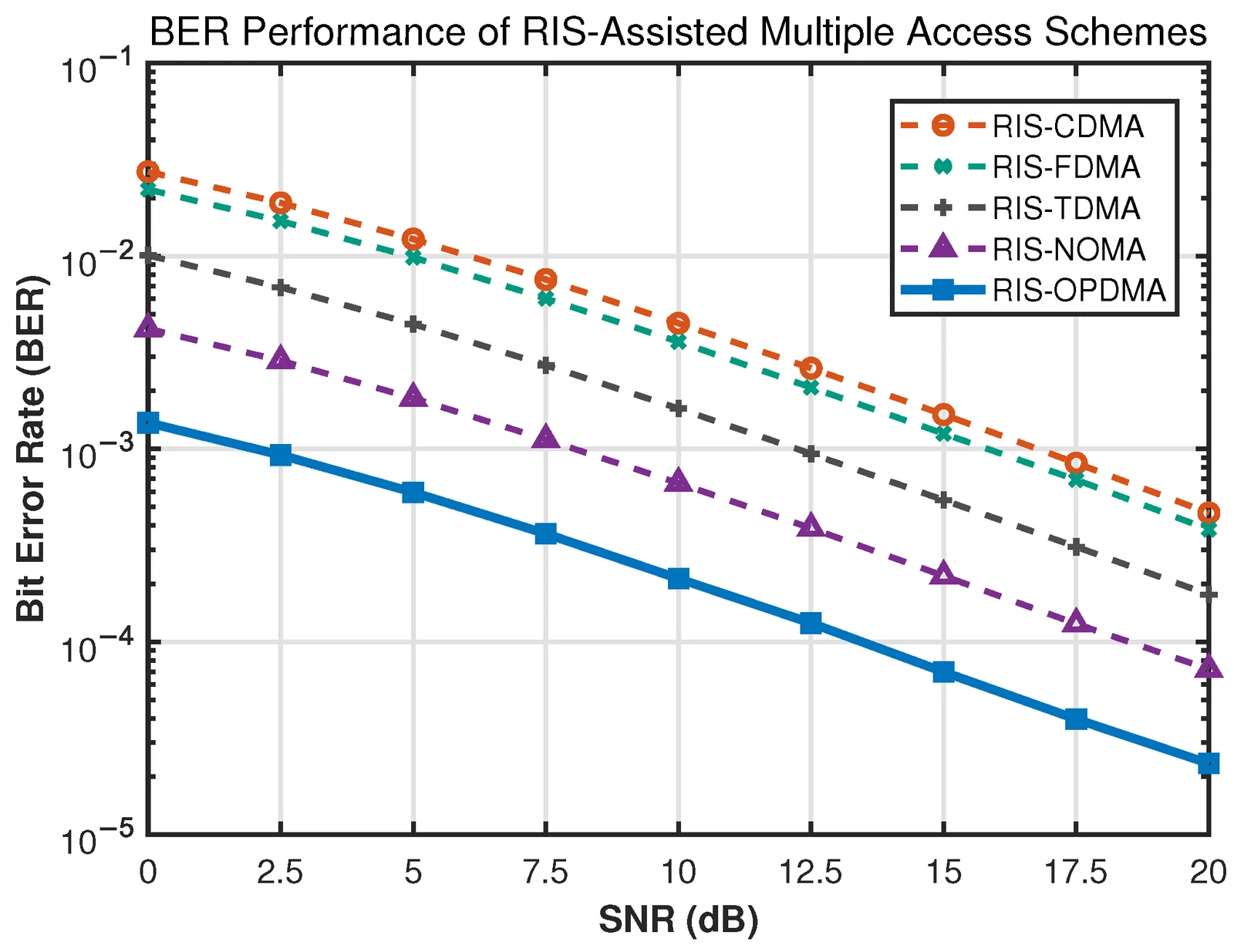
BER performance comparison of RIS-assisted multiple access schemes.

As illustrated in Figure 5, the proposed RIS-assisted two-dimensional OPDMA scheme achieves optimal BER performance across the entire SNR range of 0–20 dB, consistently outperforming RIS-CDMA, RIS-FDMA, RIS-TDMA and RIS-NOMA. Traditional time-, frequency-, and code-domain orthogonal multiple access schemes are severely affected by coupled multipath-Doppler interference introduced by RIS reflections, with performance ordered as RIS-CDMA > RIS-FDMA > RIS-TDMA. Although RIS-NOMA, a mainstream 6G non-orthogonal multiple access technique, outperforms conventional orthogonal schemes, it suffers performance degradation due to inter-user power domain interference and extra RIS-induced multipath components. Benefiting from the synergistic anti-fading mechanism where RIS mitigates large-scale shadow fading and two-dimensional OPDMA suppresses small-scale multipath and Doppler interference via optimal frequency-hopping patterns, the proposed scheme exhibits more prominent performance gains at high SNR values, verifying its reliability and robustness in complex shadowed multipath channels.

### 5.3. Throughput Performance Evaluation

On the basis of reliability evaluation from BER results, we further compare the transmission efficiency of different schemes in terms of system throughput.

**Figure 6:**
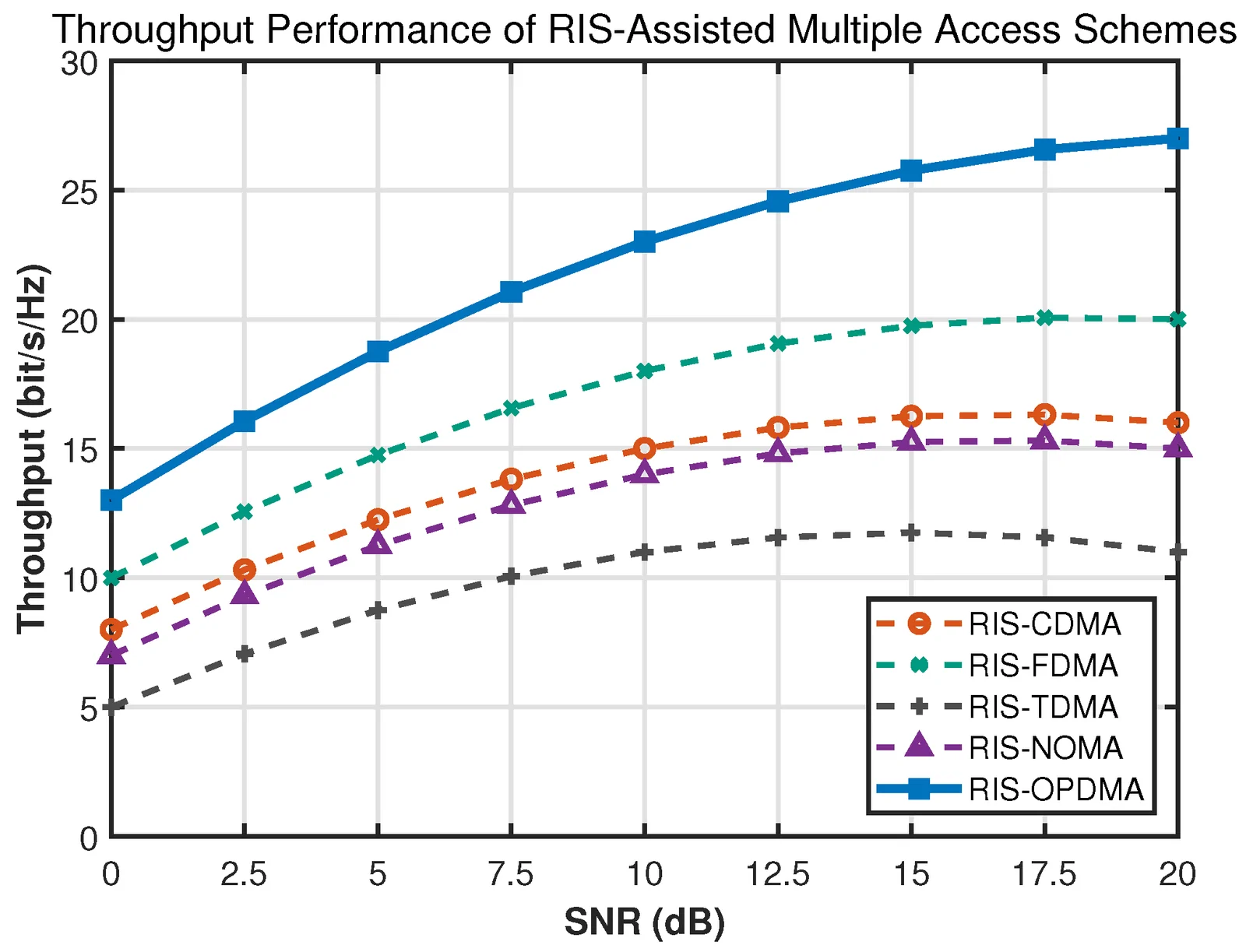
Throughput performance of RIS-assisted multiple access schemes.

As illustrated in Figure 6, the throughput performance of all RIS-assisted multiple access schemes rises progressively with increasing SNR and gradually plateaus at high SNR levels, reflecting the effective data transmission capacity of each scheme. The proposed RIS-assisted two-dimensional OPDMA scheme delivers the highest throughput within the entire 0–20 dB SNR range, surpassing RIS-FDMA, RIS-CDMA, RIS-NOMA and RIS-TDMA. Different from BER performance dominated by error probability, the throughput of traditional orthogonal multiple access schemes is limited not only by multipath–Doppler interference induced by RIS reflections but also by low spectral utilization, leading to slow growth and early saturation. Although RIS-NOMA improves spectral efficiency via power domain multiplexing, its throughput is constrained by severe inter-user interference and redundant multipath components, which reduce the proportion of successfully received data. Benefiting from the joint anti-fading mechanism where RIS alleviates large-scale shadow fading and two-dimensional OPDMA mitigates small-scale interference using optimal frequency-hopping patterns, the proposed scheme maintains high data-receiving reliability and spectral efficiency simultaneously, thus achieving superior system capacity for future 6G communication applications.

It should be noted that the performance evaluation presented in this section is based on Monte Carlo simulations, and a closed-form or approximate BER expression has not been derived. Therefore, the reported results are subject to the adopted system assumptions and simulation parameter settings. Theoretical BER analysis under more general channel conditions will be investigated in future work.

In addition, future 6G systems are expected to continue employing OFDMA, which has been widely adopted in 4G and 5G networks. However, orthogonal frequency division multiplexing (OFDM), the underlying modulation technique of OFDMA, is inherently sensitive to Doppler shifts, which destroy the orthogonality among subcarriers and result in inter-carrier interference (ICI). OPDMA has been proposed as an effective solution to address this limitation. A performance comparison between optimal frequency-hopping patterns and OFDM is provided in [23].

## 6. Conclusions

Large-scale and small-scale fading remain significant challenges in wireless communication systems. Conventional approaches to mitigating large-scale fading, such as increasing base station density or employing high-gain antennas, often incur substantial deployment costs. RIS provides a cost-effective alternative for mitigating large-scale fading; however, its deployment may introduce additional multipath propagation. Traditional techniques for combating small-scale fading generally rely on complex channel estimation and receiver equalization. In contrast, OPDMA based on optimal frequency-hopping patterns with ideal autocorrelation and cross-correlation properties effectively mitigates the effects of multipath propagation, Doppler shifts, and inter-user interference, thereby reducing the BER and improving system throughput.

Moreover, optimal frequency-hopping patterns with ideal autocorrelation and cross-correlation properties are fundamental to the design of integrated sensing and communication (ISAC) signals for future 6G systems. For sensing applications, these patterns produce a thumbtack-shaped ambiguity function and an ideal cross-ambiguity function, enabling high-resolution target detection and effective mutual interference suppression [23]. For communication applications, they enable OPDMA, improving communication reliability and overall system performance by effectively mitigating the effects of multipath propagation, Doppler shifts, and inter-user interference. Therefore, the integration of RIS and OPDMA provides a promising framework in which RIS mitigates large-scale fading, while OPDMA mitigates the effects of multipath propagation, Doppler shifts, and inter-user interference, thereby supporting efficient, reliable wireless communications and enabling future ISAC networks.

## Figures and Tables

**Table 1:** Comparison of base stations, relays, and RIS.

Feature	Base Station	Relay	RIS
Power Consumption	High	Medium	Low
Deployment Cost	High	Medium	Low
Complexity	High	Medium	Low
Coverage Extension	Limited	Moderate	High

**Table 2:** Simulation parameters for the OPDMA system.

Parameter	Value
Number of RIS Elements	64 RIS elements
BS–RIS Distance	50 m
RIS–User Distance	50 m
BS–User Distance	100 m
Cell Configuration	M=4 cells per cluster, l=4 users per cell
Doppler Shift	$f_{D}=100$ Hz, $D_{\max}=1$ (normalized)
Multipath Delay	$\tau_{\max}=2$ (normalized)
Delay Distance	Δτ=3 (minimum within families)
Modulation Scheme	QPSK (quadrature phase shift keying)
Path loss Model	Distance-dependent path loss (normalized)
Channel Model	Rayleigh fading + Log-normal shadow fading ($\sigma_{\mathrm{shadow}}=1$ dB)
RIS Phase Optimization	Select optimal phases from 2-bit codebook for maximum channel gain
Receiver Configuration	Single-antenna coherent receiver, perfect channel estimation
Monte Carlo Simulation	$10^{6}$ transmitted symbols per SNR point

## Data Availability

The original contributions presented in this study are included in the article. Further inquiries can be directed to the corresponding author.
